# Prevalence and Interrelationships of Screen Time, Visual Disorders, and Neck Pain Among University Students: A Cross-Sectional Study at Majmaah University

**DOI:** 10.3390/healthcare12202067

**Published:** 2024-10-17

**Authors:** Hind Almutairi, Layan Alhammad, Bader Aldossari, Asma Alonazi

**Affiliations:** Department of Physical Therapy and Health Rehabilitation, College of Applied Medical Sciences, Majmaah University, Al-Majmaah 11952, Saudi Arabia; hindkhaled703@gmail.com (H.A.); layann.8830@gmail.com (L.A.); bader9f@gmail.com (B.A.)

**Keywords:** students, visual disorders, neck pain, neck disability index, relationship

## Abstract

Background: Digital devices significantly contribute to vision-related problems. In addition, prolonged postural imbalance, owing to excessive use of digital devices, can lead to the tightening of head and neck muscles, resulting in neck pain, a common musculoskeletal discomfort that significantly affects individuals with poor vision. This study aimed to evaluate the prevalence and interrelationships of screen time, visual disorders, and neck pain among students at Majmaah University. Methods: A cross-sectional study was conducted at Majmaah University, Saudi Arabia, enrolling students aged 18 to 25 years. Exclusion criteria included neurological or musculoskeletal disorders. Demographic data and information on visual and neck pain symptoms were collected. The Neck Disability Index questionnaire was used to assess neck pain, and data were analyzed using SPSS version 24.0. Results: Among 263 participants, 53.6% were female. Nearsightedness (38.0%) and dry/itchy eyes (49.0%) were the most common visual disorders and symptoms, respectively. Visual disorders were prevalent in 62.0% of students, while neck pain was reported by 79.5%. Females and those studying for more than 5 h using electronic screens daily reported higher neck disability index scores. A significant association was found between >5 h of study duration [screen time] and neck disability (OR 3.703, 95% CI 1.500–9.144, *p* = 0.005). Conclusion: The study highlights a relationship between visual problems and neck pain among university students, emphasizing the need for addressing vision-related issues to reduce neck discomfort. High screen time could substantially increase the odds of developing neck disability. However, authors warrant cautious interpretation in the light of following limitations: cross-sectional study, small sample size, lack of statistical power, and self-reported data.

## 1. Introduction

Increased screen time and its abundant adverse effects on health have been widely reported in scientific literature [1,2], two being the non-vision-threatening (such as digital eye strain) [3] and vision-associated issues (such as near-sightedness) [4]. Digital devices have contributed to and exacerbated the pain, discomfort, strain, redness, and irritation of the eyes, often resulting in symptoms referred to as digital eye strain (DES) or computer vision syndrome (CVS) [5]. Continuous use of digital devices has been identified as a major contributing factor to both visual and musculoskeletal discomfort among students [6,7]. Poor posture, improper ergonomics, and extended periods of screen use led to symptoms such as neck, shoulder, and back pain, which in turn can strain the eyes [8,9]. This discomfort often causes students to adopt altered head positions that exacerbate eye strain [10]. These issues often manifest in various ways and can impact daily activities and overall quality of life.

Neck pain is the most commonly reported musculoskeletal discomfort [11] and substantially affects individuals with visual disorders like poor vision [12]. Visual disorder, postural imbalance, and neck pain and discomfort are interconnected concepts. Visual, somatosensory, and vestibular systems collectively preserve the postural balance. Poor postural balance such as hunching forward, squinting, or uneven head tilting is often triggered by misalignment in visual systems. Prolonged postural imbalance may result in tightening of head and neck muscles, resulting in neck pain and discomfort [13,14].

In the past two decades, vision-related disturbances have been frequently documented in individuals with disorders of neck pain [15,16,17]. Reportedly, jumping words, blurred vision, and inability to read with focus are common among those with neck pain disorders. Likewise, symptoms associated with disordered focal vision, like dry and itchy eyes and fatigue, are becoming a snowballing issue owing to the rising trend in the use of digital devices. In fact, the term ‘computer vision syndrome’ is now regarded as a separate domain, which could simultaneously occur in individuals with neck pain and has been implied to be a potential etiological factor of neck-related problems [18].

Given the study-related tasks and responsibilities, assumingly, students are particularly prone to disproportionate use of smart devices and high screen time. In research literature from Saudi Arabia, we did not find a current research study that investigated the prevalence of visual disorders and neck pain and their relationship among university students. Therefore, we hypothesized that high screen time, eye-related symptoms, and visual disorders would be comparatively prevalent among those with neck pain and with varied severity of neck pain in contrast to those without.

Specifically, the present study is designed and performed to achieve the following objectives: (1) to determine the prevalence of visual disorders such as near-sightedness, far-sightedness, astigmatism, etc. among students of Majmaah University, Saudi Arabia; (2) to estimate the prevalence and extent of neck pain; (3) to identify the sociodemographic and clinical factors that might have relationship with visual disorders and neck pain; and (4) to determine the factors associated with neck disability.

## 2. Materials and Methods

### 2.1. Ethics Approval

Prior to the commencement of this investigation, ethical approval was sought from the Deanship of Scientific Research, Majmaah University for Research Ethics Committee, Al Majma’ah, Saudi Arabia with Reference # MUREC-Mar.10/COM-2024-8-1. The objectives of the study were explained to prospective participants and informed consent was obtained. No identifiers were assigned to participants’ research data to maintain anonymity. Data was stored in a password-protected computer and access was only given to research team members.

### 2.2. Study Design, Setting, and Participants

A cross-sectional observational research study was conducted at Majmaah University, College of Applied Medical Sciences, Al-Majma’ah, Kingdom of Saudi Arabia to explore visual problems and neck pain among students. The present study invited both non-medical and medical students of Majmaah University. Students were enrolled via a non-probability convenience sampling technique. Neck disability index questionnaire was used to determine the prevalence and extent of neck pain [19].

The predefined selection criteria for study participants were: (1) students at Majmaah University, (2) students aged between 18 to 25 years, (3) consenting students, and (4) students with a complete neck disability index questionnaire. This study excluded students who already had a diagnosis of neurological or musculoskeletal disorders assuming that they could potentially influence both visual and neck pain symptoms independently.

### 2.3. Sociodemographic Questionnaire

Demographic and clinical characteristics of study participants were collected, including age, gender, body mass index (BMI), major, visual disorders (such as eye dryness, near-sightedness, farsightedness, amblyopia, and astigmatism), eye-related symptoms (including dryness, itchiness, redness, and pressure), study duration (less than 2 h, 2–5 h, and more than 5 h), and types of smart devices used.

### 2.4. Neck Disability Index Questionnaire

The neck disability index questionnaire is a self-reporting tool designed to measure the effect of neck pain on activities of daily living. The questionnaire typically consists of ten items, each addressing different aspects of daily life and activities affected by neck pain. Respondents rate the extent of their pain on a scale from 0 to 5 for each item, with 0 indicating no pain and 5 indicating complete disability due to pain. The total score is calculated by summing the individual item scores, and the maximum possible score is 50. The scoring for interpretation consists of the following intervals: 0–4 points (no pain/disability), 5–14 points (mild limitation/disability), 15–24 points (moderate limitation/disability), 25–34 (severe limitation/disability), and >34 points (complete activity limitation/disability). The higher the total score, the greater the activity of daily living is impacted. The lower the score, the lesser the limitations towards the performance of daily activities. The reported test–retest reliability is extremely high, i.e., intraclass correlation of 0.96 [19].

### 2.5. Outcome Measures

The key outcome measures of this research investigation were: (1) prevalence of visual disorders such as near-sightedness, far-sightedness, astigmatism, etc., among students of Majmaah University, Saudi Arabia, (2) prevalence and extent of neck pain, (3) relationship of sociodemographic and clinical factors (including screen time) with visual disorders and neck pain, (4) relationship between visual disorders and neck pain, and (5) determination of factors associated neck disability.

### 2.6. Statistical Analyses

Microsoft Excel Office 365 was used for initial data entry and management. Subsequently, Statistical Package for Social Sciences (SPSS) for Windows, version 24.0 (IBM Corp., Armonk, NY, USA) was utilized for data analyses. Depending on data distribution, quantitative variables were presented as means ± standard deviations (SDs) or median and interquartile range (IQR), and qualitative variables were expressed as numbers and percentages. Mean differences in neck disability index scores were computed using independent samples *t*-test or One-way ANOVA, depending on data dynamics. Similarly, the Chi-Square test or Fisher’s Exact test was performed to determine, if any, the relation between the sociodemographic characteristics of participants and neck disability index scores. Logistic regression analysis was performed and odds ratios (ORs) with their respective 95% confidence intervals (CIs) were reported. For all statistical tests, *p*-value < 0.05 was set as statistically significant.

## 3. Results

### 3.1. Sociodemographic and Clinical Characteristics

Overall, 263 students participated and completed the entire questionnaire. Female participation was slightly predominant compared to males (*n* = 141, 53.6%). The mean BMI was 23.04 ± 4.92 kg/m^2^ i.e., over half of the participants had normal BMI (*n* = 148, 56.3%). Participation from both non-medical and medical specialty students was almost equal (53.2% versus 46.8%). In terms of visual disorders, near-sightedness was common in over one-third of the participants (*n* = 100, 38.0%), followed by far-sightedness (*n* = 35, 13.3%) and astigmatism (*n* = 32, 12.2%). Among eye-related symptoms, dry/itchy eyes (*n* = 129, 49.0%) and pressure in the eye/forehead area (*n* = 79, 30.0%) were frequently noted. Of 263 participants, 33.8% (*n* = 89) reported studying for >5 h. The iPad was by far the most frequently used smart device among students (*n* = 197, 74.9%). Table 1 displays the sociodemographic and clinical characteristics of the participating students.

### 3.2. Prevalence of Visual Disorders, Eye-Related Symptoms, and Neck Pain

Visual disorders were present in 62.0% (*n* = 163), eye-related symptoms in 78.9% (*n* = 189), and neck pain in 79.5% (*n* = 209) of participants. The mean neck pain disability index score was found to be 11.90 ± 8.18. The interpretation of the neck pain disability index score revealed that the majority of the patients either had no pain (*n* = 54, 20.5%) or mild (*n* = 114, 43.3%) to moderate (*n* = 77, 29.3%) disability (Table 1).

### 3.3. Differences in Neck Disability Index Scores Based on Sociodemographic and Clinical Characteristics

Upon studying the mean or median differences in neck disability index scores for sociodemographic and clinical characteristics of study participants (Table 2), we found that females (14.55 ± 8.08 versus 8.85 ± 7.20, *p* < 0.0001), those studying >5 h (13.33 ± 8.14 versus 9.50 ± 9.09 versus 11.93 ± 7.58, *p* = 0.025), those who had eye-related symptoms (13.17 ± 8.39 versus 9.84 ± 7.40, *p* = 0.001), and those with visual disorders (13.13 ± 8.47 versus 8.77 ± 6.44, *p* < 0.0001) were significantly associated with higher neck disability index scores compared to others. No differences in neck disability index scores were observed for body mass index (*p* = 0.300), specialty (*p* = 0.300), and use of specific smart devices (*p* = 0.220).

### 3.4. Relationship between Sociodemographic and Clinical Characteristics and Visual Disorders

Table 3 presents the results of the relationship between sociodemographic and clinical characteristics and visual disorders. Only the gender variable was found to be associated with visual disorders, with females reporting visual disorders more frequently compared to that of males (58.9% versus 41.1%, *p* = 0.028). We did not find any relationship between body mass index (*p* = 0.378), specialty (*p* = 0.481), study duration (*p* = 0.788), or use of specific smart devices (*p* = 0.587); however, a trend was witnessed confirming that visual disorders were comparatively more common in those with neck pain (82.2% versus 75.5%, *p* = 0.160) than without.

### 3.5. Relationship between Sociodemographic and Clinical Characteristics and Extent of Visual Disorders

Table 4 illustrates the differences in the frequency of extent of neck pain (categorized by no pain, mild disability, moderate disability, severe disability, and complete disability) based on diverse sociodemographic and clinical characteristics. Females were found to be statistically more inclined to possess mild disability (53.5% versus 46.5%), moderate disability (67.5% versus 32.5%), severe disability (87.5% versus 12.5%), and complete disability (50.0% versus 50.0%) compared to males (*p* < 0.0001) and the three Rs of waste management (reduce, reuse, recycle) (86.9% versus 74.7%, *p* = 0.004). Those studying either 2–5 h or >5 h demonstrated a higher frequency of mild disability to complete disability compared to that of those who studied <2 h (mild disability: 47.4% and 33.3% versus 19.3%, moderate disability: 49.4% and 44.2% versus 6.5%, severe disability: 43.8% and 31.3% versus 25.0%, and complete disability: 0.0% and 50.0% versus 50.0%, *p* < 0.0001). Similarly, students who were more disposed to using iPads as smart devices had higher occurrences of neck-related disability compared to those using smartphones or laptops (mild disability: 75.4% versus 6.1% and 18.4%, moderate disability: 79.2% versus 14.3% and 6.5%, severe disability: 81.3% versus 18.8% and 0.0%, and complete disability: 50.0% versus 50.0% and 0.0%, *p* = 0.006). Statistical differences for prevalence of neck-related disability were higher and more significant in those with visual disorders (mild disability: 56.1% versus 43.9%, moderate disability: 72.7% versus 27.3%, severe disability: 81.3% versus 18.8%, and complete disability: 50.0% versus 50.0%, *p* = 0.031) and eye-related symptoms (mild disability: 65.8% versus 34.2%, moderate disability: 85.7% versus 14.3%, severe disability: 93.8% versus 6.3%, and complete disability: 100.0% versus 0.0%, *p* < 0.0001) compared to that of their counterparts.

### 3.6. Factors Associated with Neck Disability

Logistic regression analysis was performed to identify factors associated with neck disability. Univariate logistic regression analysis revealed the following statistically significant factors: female gender (OR 4.984, 95% CI 2.517–9.868, *p* < 0.0001), having eye-related symptoms (OR 2.299, 95% CI 1.230–4.294, *p* = 0.009), >5 h of study duration [screen time] (OR 4.875, 95% CI 2.120–11.208, *p* < 0.0001), and use of iPad smart device (OR 2.643, 95% CI 1.217–5.736, *p* = 0.014). On multivariate logistic regression analysis, >5 h of study duration [screen time] remained statistically significant, demonstrating nearly four times higher odds of individuals presenting with neck disability (OR 3.703, 95% CI 1.500–9.144, *p* = 0.005).

## 4. Discussion

This study was conducted to evaluate the prevalence and interrelationships of screen time, visual disorders, and neck disability among students of Majmaah University in Al-Majma’ah, Saudi Arabia. Our findings confirmed that individuals with neck pain and disability of varied severity had a substantially greater prevalence of eye-related symptoms and visual disorders. Reported eye-related symptoms were dry/itchy eyes, redness in the eye, and pressure in the eye/forehead area; visual disorders were near-sightedness, far-sightedness, amblyopia, and astigmatism. Our findings align with a study conducted in Pakistan by Shabbir et al. (2022), which reported a statistically significant correlation between near-sightedness (myopia) and neck pain [14]. Similarly, a case-control study from Australia by Treleaven and Takasaki (2014) indicated a higher prevalence of visual disturbances (such as visual fatigue, eye strain, light sensitivity, heavy eyes, and difficulty concentrating while reading) among individuals with neck pain compared to those without symptoms, with severity more pronounced in cases of traumatic neck pain [20].

Neck pain is one of the most frequently reported symptoms of the musculoskeletal system in the general population. The age-standardized prevalence rate of neck pain was 27/1000 of the population in 2019 [21]. While several risk factors have been documented in the literature [21], visual disorders have gained significant traction lately due to the increased use of electronic gadgets [22]. From a pathophysiological perspective, conditions such as near-sightedness (myopia) lead to the contraction of orbicularis oculi muscle, resulting in squinting to focus the eyes. Prolonged contraction can disturb tear film, corneal health, and eyelid function, potentially triggering symptoms in the neck region. Poor vision can also trigger asymmetrical use of neck muscles owing to inappropriate head-to-trunk orientation, affecting overall postural balance. Prolonged adaptation to inappropriate head positions, such as forward head posture, can lead to stiffness in the head and neck muscles, ultimately contributing to neck pain and disability [14].

Our findings also demonstrated higher odds of neck disability among those with high screen time for their study. Pirnes et al. (2022) have demonstrated a significant association between overall screen time and neck and shoulder pain in school-aged children [23]. A recent systematic review and meta-analysis by Gao et al. (2023) also corroborated the association of high electronic device usage (OR = 1.53, 95% CI: 1.33 to 1.76) with neck pain among college students [24]. Mechanistically, excessive use of electronic devices, such as computers, can lead to continuous staring at monitors with a forward head position, which exaggerates the anterior curve in the lower cervical vertebrae and the posterior curve in the upper thoracic vertebrae, resulting in forward head posture [25]. Visual disorders, such as near-sightedness, have also been correlated with forward head posture [26]. A study by Lee et al. (2021) outlined how laptop and tablet use can alter neck flexion, bilateral shoulder elevation, and flexion of the upper trunk in contrast to a desktop computer. They recommended that people with chronic neck pain must be wary of their posture, particularly with hand-held electronic devices [27]. Shin et al. (2017) similarly demonstrated elevated neck pain with alteration in scapular muscle alignment among young women with forward head posture [28]. Considering that students are exposed to smart devices and high screen time due to academic and personal use; our findings and the reviewed literature provide valuable insights.

Another key finding in the present study was gender-related differences in neck pain, with females reporting higher neck disability index scores compared to male students. This pattern has been observed in previous studies, where more neck-related complaints were reported by female participants [29,30]. We also observed a significantly higher prevalence of visual disorders among females (58.9% versus 41.1%). Thus, based on its effect on neck muscles, this could potentially influence neck pain in our study participants.

While there is no relevant literature regarding the mental health hazards of high screen time, visual disturbance, and neck pain, it is worth discussing their plausible link. Visual disturbances, like eye strain due to high exposure to electronic screens, may cause compensatory alterations in posture; for instance, forward head posture, which can aggravate the risk of developing neck pain. This can be expected among university students who regularly engage in long study hours using electronic devices. Persistence of the aforementioned physical hazards might deteriorate mental well-being, as extended discomfort can cause high stress, anxiety, and diminished academic performance.

The findings of our research study are limited by a few factors that warrant discussion, hence results must be carefully interpreted and generalized in the light of these limitations. This descriptive, cross-sectional study had a small sample size with convenience sampling, which may limit the generalization of study conclusions, hinder establishing visual disorders as a causal risk factor, and introduce selection bias. Additionally, the lack of presentation of statistical power and effect size values means we cannot fully assess the robustness of our findings or the magnitude of the observed effects. Also, the use of self-reported data may have biased the findings and should be noted. It should also be noted that the present study did not take into account other variables such as stress, physical activity, or pre-existing conditions, which could possibly infer neck discomfort and visual complaints. However, greater control for these confounders would have increased the quality of the study. Importantly, our study only considered self-reported visual disorders and did not account for other aspects of oculomotor function like smooth pursuit, saccade accuracy, eye vergence, and other daily errands that are visually challenging. Furthermore, this study did not account for the use of spectacles. Appropriately fitted glasses can alleviate visual issues; therefore, this might have influenced our results. Another limitation was that we did not determine the presence or absence of co-existing diseases of somatosensory and vestibular systems. It has been communicated that visual, somatosensory, and vestibular systems jointly function to maintain postural balance, and disruption in any of these systems could lead to neck musculoskeletal issues [13,14]. Finally, the mechanistic relationship between visual disorders and neck and scapular muscle adjustment was not explored.

## 5. Conclusions

In conclusion, the findings of the present study confirm that eye-related symptoms and visual disorders in students could lead to mild and moderate to severe and complete neck pain. In fact, our findings also suggest that studying for 2 to 5 h or >5 h and using an iPad smart device could substantially increase the severity of neck pain. High screen time was associated with increased odds of developing neck disability. Visual, somatosensory, and vestibular systems work in alliance to preserve postural balance. Misalignment in any of the systems could trigger postural imbalance. With the conceivable elucidation of the relationship among visual disorders, poor postural balance, and neck discomfort in scientific literature, our findings emphasize the importance of correcting vision-related problems, if present, in individuals with neck musculoskeletal issues. Finally, to improve its practical impact, universities must consider implementing awareness sessions on proper posture and screen use and develop policies to manage screen time among students.

## Figures and Tables

**Table 1:** Sociodemographic and Clinical Characteristics of Study Participants (*n* = 263).

Demographic Variables	Frequency (%)
Age (Years, Mean ± SD)	20.71 ± 1.74
Gender	
Female	141 (53.6%)
Male	122 (46.4%)
Body Mass Index (kg/m^2^, Mean ± SD)	23.04 ± 4.92
Normal	148 (56.3%)
Underweight	44 (16.7%)
Overweight	42 (16.0%)
Obese	29 (11.0%)
Specialty	
Non-Medical	140 (53.2%)
Medical	123 (46.8%)
Visual Disorders	
None	100 (38.0%)
Eye Dryness	2 (0.8%)
Near Sightedness	100 (38.0%)
Far Sightedness	35 (13.3%)
Amblyopia	2 (0.8%)
Astigmatism	32 (12.2%)
Eye-related Symptoms	
None	74 (28.1%)
Dry/Itchy Eyes	129 (49.0%)
Redness in the Eye	57 (21.7%)
Pressure in the Eye/Forehead Area	79 (30.0%)
Study Duration (Screen Time)	
<2 h	54 (20.5%)
2–5 h	120 (45.6%)
>5 h	89 (33.8%)
Type of Smart Device	
Smart Phone	35 (13.3%)
iPad	197 (74.9%)
Laptop	31 (11.8%)
Neck Pain Disability Index Score	11.90 ± 8.18
Neck Disability Index	
No Disability	54 (20.5%)
Mild	114 (43.3%)
Moderate	77 (29.3%)
Severe	16 (6.1%)
Complete	2 (0.8%)

Abbreviation: SD, Standard Deviation.

**Table 2:** Sociodemographic and Clinical Characteristics and Neck Disability Index Scores (*n* = 263).

Variables	Neck Disability Index (Mean ± SD or Median IQR)	*p* Value
Gender		
Female	14.55 ± 8.08	<0.0001
Male	8.85 ± 7.20	
Body Mass Index		
Normal	12.00 (6.00–18.75)	0.300 ^†^
Underweight	8.50 (4.25–17.00)	
Overweight	11.50 (5.75–16.00)	
Obese	8.00 (6.50–12.50)	
Specialty		
Non-Medical	12.39 ± 8.54	0.300
Medical	11.35 ± 7.74	
Study Duration (Screen Time)		
<2 h	9.50 ± 9.09	0.025
2–5 h	11.93 ± 7.58	
>5 h	13.33 ± 8.14	
Smart Device		
Smart Phone	10.00 (4.00–20.00)	0.220 ^†^
iPad	12.00 (6.00–17.00)	
Laptop	9.00 (5.00–14.00)	
Visual Disorders		
No Disorders	9.84 ± 7.40	0.001
Visual Disorders	13.17 ± 8.39	
Eye-related Symptoms		
No Symptoms	8.77 ± 6.44	<0.0001
Eye-related Symptoms	13.13 ± 8.47	

Abbreviations: SD, Standard Deviation. IQR, Interquartile Range. **^†^** Kruskal-Wallis H Test.

**Table 3:** Sociodemographic and Clinical Characteristics and Visual Disorders (*n* = 263).

Variables	No Disorders (*n* = 100)	Visual Disorders (*n* = 163)	*p* Value
Gender			
Female	45 (45.0%)	96 (58.9%)	0.028
Male	55 (55.0%)	67 (41.1%)	
Body Mass Index			
Normal	50 (50.0%)	98 (60.1%)	0.378
Underweight	20 (20.0%)	24 (14.7%)	
Overweight	19 (19.0%)	23 (14.1%)	
Obese	11 (11.0%)	18 (11.0%)	
Specialty			
Non-Medical	56 (56.0%)	84 (51.5%)	0.481
Medical	44 (44.0%)	79 (48.5%)	
Study Duration (Screen Time)			
<2 h	22 (22.0%)	32 (19.6%)	0.788
2–5 h	43 (43.0%)	77 (47.2%)	
>5 h	35 (35.0%)	54 (33.1%)	
Smart Device			
Smart Phone	16 (16.0%)	19 (11.7%)	0.587
iPad	72 (72.0%)	125 (76.7%)	
Laptop	12 (12.0%)	19 (11.7%)	
Neck Disability			
No Disability	25 (25.0%)	29 (17.8%)	0.160
Neck Disability	75 (75.0%)	134 (82.2%)	

**Table 4:** Sociodemographic and Clinical Characteristics and Extent of Neck Pain (*n* = 263).

Neck Disability Index	No Disability (*n* = 54)	Mild (*n* = 114)	Moderate (*n* = 77)	Severe (*n* = 16)	Complete (*n* = 2)	*p* Value
Gender						
Female	13 (24.1%)	61 (53.5%)	52 (67.5%)	14 (87.5%)	1 (50.0%)	<0.0001 ^†^
Male	41 (75.9%)	53 (46.5%)	25 (32.5%)	2 (12.5%)	1 (50.0%)	
Body Mass Index						
Normal	28 (51.9%)	64 (56.1%)	43 (55.8%)	11 (68.8%)	2 (100.0%)	0.846 ^†^
Underweight	11 (20.4%)	17 (14.9%)	13 (16.9%)	3 (18.8%)	0 (0.0%)	
Overweight	9 (16.7%)	16 (14.0%)	15 (19.5%)	2 (12.5%)	0 (0.0%)	
Obese	6 (11.1%)	17 (14.9%)	6 (7.8%)	0 (0.0%)	0 (0.0%)	
Specialty						
Non-Medical	27 (50.0%)	62 (54.4%)	40 (51.9%)	9 (56.3%)	2 (100.0%)	0.824 ^†^
Medical	27 (50.0%)	52 (45.6%)	37 (48.1%)	7 (43.8%)	0 (0.0%)	
Study Duration (Screen Time)						
<2 h	22 (40.7%)	22 (19.3%)	5 (6.5%)	4 (25.0%)	1 (50.0%)	<0.0001 ^†^
2–5 h	21 (38.9%)	54 (47.4%)	38 (49.4%)	7 (43.8%)	0 (0.0%)	
>5 h	11 (20.4%)	38 (33.3%)	34 (44.2%)	5 (31.3%)	1 (50.0%)	
Smart Device						
Smart Phone	13 (24.1%)	7 (6.1%)	11 (14.3%)	3 (18.8%)	1 (50.0%)	0.006 ^†^
iPad	36 (66.7%)	86 (75.4%)	61 (79.2%)	13 (81.3%)	1 (50.0%)	
Laptop	5 (9.3%)	21 (18.4%)	5 (6.5%)	0 (0.0%)	0 (0.0%)	
Visual Disorders						
No Disorders	25 (46.3%)	50 (43.9%)	21 (27.3%)	3 (18.8%)	1 (50.0%)	0.031 ^†^
Visual Disorders	29 (53.7%)	64 (56.1%)	56 (72.7%)	13 (81.3%)	1 (50.0%)	
Eye-related Symptoms						
No Symptoms	23 (42.6%)	39 (34.2%)	11 (14.3%)	1 (6.3%)	0 (0.0%)	<0.0001 ^†^
Eye-related Symptoms	31 (57.4%)	75 (65.8%)	66 (85.7%)	15 (93.8%)	2 (100.0%)	

Note: ^†^ Fisher’s Exact Test.

## Data Availability

For any queries or requests regarding the data, please contact [Corresponding Author: Asma Alonazi] at [a.alonazi@mu.edu.sa].

## References

[1] Nakshine V.S., Thute P., Khatib M.N., Sarkar B. (2022). Increased Screen Time as a Cause of Declining Physical, Psychological Health, and Sleep Patterns: A Literary Review. Cureus.

[2] Alhaque Roomi M.H.A., Srivastava A., Girdhar N., Jha C., Thakur S. (2024). A Study of the Correlation between Screen Time and Hypertension among Young Adults in North India: A Cross-Sectional Analysis. Cureus.

[3] Agarwal R., Tripathi A., Khan I.A., Agarwal M. (2022). Effect of increased screen time on eyes during COVID-19 pandemic. J. Fam. Med. Prim. Care.

[4] Champagne-Hamel M., Monfort C., Chevrier C., Saint-Amour D. (2023). Screen Time at 6 Years Old and Visual Function in Early Adolescence. Vision.

[5] Pavel I.A., Bogdanici C.M., Donica V.C., Anton N., Savu B., Chiriac C.P., Pavel C.D., Salavastru S.C. (2023). Computer Vision Syndrome: An Ophthalmic Pathology of the Modern Era. Medicina.

[6] Tsang S.M.H., Cheing G.L.Y., Lam A.K.C., Siu A.M.H., Pang P.C.K., Yip K.-C., Chan J.W.K., Jensen M.P. (2023). Excessive use of electronic devices among children and adolescents is associated with musculoskeletal symptoms, visual symptoms, psychosocial health, and quality of life: A cross-sectional study. Front. Public Health.

[7] Straker L., Harris C., Joosten J., Howie E.K. (2018). Mobile technology dominates school children’s IT use in an advantaged school community and is associated with musculoskeletal and visual symptoms. Ergonomics.

[8] Devi K.A., Singh S.K. (2023). The hazards of excessive screen time: Impacts on physical health, mental health, and overall well-being. J. Educ. Health Promot..

[9] Sánchez-González M.C., Gutiérrez-Sánchez E., Sánchez-González J., Rebollo-Salas M., Ruiz-Molinero C., Jiménez-Rejano J.J., Pérez-Cabezas V. (2019). Visual system disorders and musculoskeletal neck complaints: A systematic review and meta-analysis. Ann. N. Y. Acad. Sci..

[10] Computer Vision Syndrome. https://www.aoa.org/healthy-eyes/eye-and-vision-conditions/computer-vision-syndrome?sso=y.

[11] Khired Z. (2022). The Prevalence of and Factors Associated with Neck Pain among Jazan Adult Population. Cureus.

[12] Zetterlund C., Lundqvist L.-O., Richter H.O. (2009). The Relationship between Low Vision and Musculoskeletal Complaints. A Case Control Study between Age-related Macular Degeneration Patients and Age-matched Controls with Normal Vision: J. Optom..

[13] Naipal S., Rampersad N. (2018). A review of visual impairment. Afr. Vis. Eye Health.

[14] Shabbir S., Sadaqat A., Wakeel M., Arslan H.R.M. (2022). Association between Visual Impairment and Neck Pain in Computer Users; A Cross-Sectional Study: Visual Impairment and Neck Pain in Computer Users. Pak. BioMed. J..

[15] Treleaven J., Jull G., LowChoy N. (2005). Smooth pursuit neck torsion test in whiplash-associated disorders: Relationship to self-reports of neck pain and disability, dizziness and anxiety. J. Rehabil. Med..

[16] Treleaven J. (2017). Dizziness, unsteadiness, visual disturbances, and sensorimotor control in traumatic neck pain. J. Orthop. Sports Phys. Ther..

[17] Daly L., Giffard P., Thomas L., Treleaven J. (2018). Validity of clinical measures of smooth pursuit eye movement control in patients with idiopathic neck pain. Musculoskelet. Sci. Pract..

[18] Teo C., Giffard P., Johnston V., Treleaven J. (2019). Computer vision symptoms in people with and without neck pain. Appl. Ergon..

[19] Shaheen A.A.M., Omar M.T.A., Vernon H. (2013). Cross-cultural adaptation, reliability, and validity of the Arabic version of neck disability index in patients with neck pain. Spine.

[20] Treleaven J., Takasaki H. (2014). Characteristics of visual disturbances reported by subjects with neck pain. Man. Ther..

[21] Kazeminasab S., Nejadghaderi S.A., Amiri P., Pourfathi H., Araj-Khodaei M., Sullman M.J.M., Kolahi A.A., Safiri S. (2022). Neck pain: Global epidemiology, trends and risk factors. BMC Musculoskelet. Disord..

[22] Alturaiki H.M., Alnajjar J.S., Alibrahim I.A., Almuhaysin F.A., El Gaddafi M.W., Almarzoq M.A., Alturaiki F.M., Aleid S.S. (2023). Computer Vision Syndrome among the General Population in the Eastern Region of Libya: Prevalence and Risk Factors. Cureus.

[23] Pirnes K.P., Kallio J., Hakonen H., Hautala A., Häkkinen A.H., Tammelin T. (2022). Physical activity, screen time and the incidence of neck and shoulder pain in school-aged children. Sci. Rep..

[24] Gao Y., Chen Z., Chen S., Wang S., Lin J. (2023). Risk factors for neck pain in college students: A systematic review and meta-analysis. BMC Public Health.

[25] Stincel O.R., Oravitan M., Pantea C., Almajan-Guta B., Mirica N., Boncu A., Avram C. (2023). Assessment of Forward Head Posture and Ergonomics in Young IT Professionals—Reasons to Worry?. Med. Lav..

[26] Islamiatun Z.K., Naufal A.F., Zulfatirrohman A.I. (2023). The Relationship between Nearsightedness and forward Head Posture. Proceedings of the International Conference on Health and Well-Being (ICHWB 2022).

[27] Lee R., James C., Edwards S., Snodgrass S.J. (2021). Posture during the use of electronic devices in people with chronic neck pain: A 3D motion analysis project. Work.

[28] Shin Y.J., Kim W.H., Gil Kim S. (2017). Correlations among visual analogue scale, neck disability index, shoulder joint range of motion, and muscle strength in young women with forward head posture. J. Exerc. Rehabil..

[29] Tsunoda D., Iizuka Y., Iizuka H., Nishinome M., Kobayashi R., Ara T., Yamamoto A., Takagishi K. (2013). Associations between neck and shoulder pain (called katakori in Japanese) and sagittal spinal alignment parameters among the general population. J. Orthop. Sci..

[30] Takasawa E., Yamamoto A., Kobayashi T., Tajika T., Shitara H., Ichinose T., Mieda T., Iizuka Y., Iizuka H., Takagishi K. (2015). Characteristics of neck and shoulder pain in the Japanese general population. J. Orthop. Sci..

